# The Treatment of Puerperal Fever by Formalin

**Published:** 1903-02-21

**Authors:** 


### THE TREATMENT OF PUERPERAL FEVER BY FORMALIN.

Much interest has lately been excited by the report of a case of puerperal fever in which recovery followed the subcutaneous injection of a considerable quantity of a weak solution of formalin. The case was a very severe one ; there seems to have been no doubt about the infection of the blood with streptococci, and the recovery followed so smartly upon the injection of the second dose of the antiseptic that the physician in attendance, Dr. Charles C. Burrows, of the Bellevue Hospital, New York, felt that the cure must be attributed to the treatment. Since this case, which occurred in December, 1902, several others have been reported in which similar treatment has been undertaken with varying results, and it is hardly to be wondered at that at the present moment opinion is much divided on the subject. The optimists hold, and with a certain show of reason, that a case of this kind, taken in conjunction with the results claimed by Dr. Maguire in regard to the intravenous administration of germicide solutions in tuberculosis, opens up a large and hopeful field for the direct treatment of blood-infections, while the more cautious doubt. This is not the first time that they have heard of wonderful cures of puerperal fever which have ended in nothing, and some go so far as to question whether the case may not after all have been one of sapræmia rather than of true infection of the blood by streptococci, and it is on this point that the whole question seems to hinge. The patient was a negro woman who had been confined eight days before, and had when admitted a temperature of 104·3° Fahr., which by the next day had risen to 107°, and it is stated that the blood on being examined bacteriologically showed the presence of streptococci. On this an intravenous injection of 500 cubic centimetres of fomalin solution, of the strength of 1 in 5,000, was made into the arm, and the next day the temperature had fallen to 101° F. The next day the temperature began to rise again, and on the third day after the injection it reached 103° F. when a second injection of 750 cubic centimetres of a similar solution was given. After this the temperature fell and the woman has remained well. It is stated, however, that the uterus in this case had very recently been cleared out when the formalin treatment was commenced, and that any good effect arising from this local treatment might be expected to show itself about the time when the amelioration of symptoms did actually take place. In any case, as it seems to us, one cannot come to any conclusion on the strength of one or two cases—certainly not to a conclusion in favour of the treatment suggested. It is not in accordance with ordinary laboratory experience to expect a well defined and demonstrable septicæmia to be brought to a stand by the injection of any chemical antiseptic; the experiment has been tried again and again.

